# Mental health of victims of sexual violence in eastern Congo: associations with daily stressors, stigma, and labeling

**DOI:** 10.1186/1472-6874-14-106

**Published:** 2014-09-06

**Authors:** An Verelst, Maarten De Schryver, Eric Broekaert, Ilse Derluyn

**Affiliations:** 1 Department of Social Welfare Studies & Centre for Children in Vulnerable Situations, Ghent University, Henri Dunantlaan 2, Ghent 9000, Belgium; 2 Department of Experimental Clinical and Health Psychology, Ghent University, Henri Dunantlaan 2, Ghent 9000, Belgium; 3 Department of Orthopedagogics, Ghent University, Henri Dunantlaan 2, Ghent 9000, Belgium

**Keywords:** War, Mental health, Adolescent girls, Stigmatization, Daily stressors, Labeling rape

## Abstract

**Background:**

The conflict-ridden context of eastern Congo has set the scene for grueling human rights violations, with sexual violence as one of the ‘weapons of war’. Currently, sexual violence continues, with a considerable increase in civilian perpetrators. However, little is known regarding the particular impact of different experiences of sexual violence on adolescents’ mental health. This study therefore investigates the impact of sexual violence on eastern Congolese adolescents’ mental health and its differing associations with daily stressors, stigma, and the labeling of sexual violence (as ‘rape’ or ‘non-consensual sexual experience’).

**Methods:**

A cross-sectional, population-based survey design was implemented in 22 secondary schools, randomly selected from a stratified sample, in Bunia, eastern Congo, a region extensively affected by war. A total of 1,305 school-going adolescent girls aged 11 to 23 participated. Self-report measures of mental health symptoms, war-related traumatic events, experiences of sexual violence, daily stressors, and stigmatization were administered. Differences in sociodemographic characteristics, traumatic experiences and daily and social stressors between types of sexual violence (rape, non-consensual sexual violence, no sexual violence) were explored through statistical analysis. ANCOVA analyses investigated associations between those risk factors and adolescents’ mental health.

**Results:**

More than one third of eastern Congolese adolescent girls reported experiences of sexual violence. Elevated levels of daily stressors, experiences of stigmatization, and stressful war-related events were found amongst girl victims of sexual violence, with the highest levels for girls who labeled the sexual violence as rape. Daily stressors, stigmatization, and war-related events showed a large impact on the girls’ mental health. Last, girls who labeled the sexual violence as non-consensual sexual experiences reported more post-traumatic hyper-arousal and intrusion symptoms compared to those labeling the sexual violence as rape.

**Conclusions:**

These findings point to the important association between how war-affected adolescent girls label sexual violence (rape or non-consensual sexual experiences) and their mental health. This study also documents the large impact of sexual violence on other stressors (daily stressors, stigmatization, and stressful war events) and the impact of these stressors on girl victims’ mental health. It discusses important implications for addressing sexual violence and its consequences in war-affected contexts.

## Background

Armed conflicts in the eastern Democratic Republic of Congo (DRC) have caused over five million casualties and displaced hundreds of thousands of civilians in the past two decades [1], with a devastating ongoing impact that persists today [2]. During these wars, the civilian population has been a primary target through, amongst other war strategies, looting, brutal massacres, torture, sexual violence, and cannibalism [3,4].

The tactic that has heightened worldwide attention to the Congolese wars is sexual violence, a ‘weapon of warfare’ [5,6] that has been strategically used in numerous conflicts throughout history [7-9] to humiliate, dominate, instill fear in, disperse, and/or forcibly relocate civilian members of communities and ethnic groups [10]. Though exact numbers on the prevalence of sexual violence in the region do not exist, there are overall estimates of nearly 1.69 to 1.80 million eastern Congolese women aged 15 to 49 years who report histories of being raped [11]). Not only are individuals seriously harmed, but also the social tissue that holds society together is torn, communities and families are destroyed and demoralized [12,13], and cultural and political solidarity is undermined [14]. In eastern DRC, sexual violence has been used in armed conflict with extreme brutality and destructiveness [5,11,14,15]. Recent reports also shed light on other dimensions of sexual violence in this war-ridden environment. A considerable increase in sexual violence perpetrated by civilians (as opposed to military personnel) and in sexual violence against minors [16,17] has been observed in these regions, with various reports and studies alluding to a ‘normalization’ or ‘civilization’ of rape in eastern Congo [5]. While officially the signing of peace agreements has brought the armed conflict in eastern Congo to an end, eastern Congolese communities perceive their region as remaining in a continuous state of war, with ongoing sexual violence as one of the principal indicators [2]. These evolutions call for a broader perspective on sexual violence in these warring contexts, since the singular discourse of ‘rape as a weapon of war’ systematically neglects other forms of sexual violence, such as domestic sexual violence and sexual violence perpetrated by civilians [2,18].

The consequences of sexual violence for victims’ mental health have been extensively described [19-21], although evidence of the impact of sexual violence, in particular on adolescents, remains limited [22]. In addition, studies have tried to identify factors impacting on these mental health consequences. Recent research has widened the scope from a dose-effect study of traumatic exposure on posttraumatic stress symptoms [23] to a broader model that includes daily stressors as important factors influencing mental health [24-27]. Daily stressors encompass both current difficult material and situational living circumstances (e.g., unemployment, disease, poverty, poor housing) and social stressors. Social stressors are widespread in community reactions to sexual violence in war-affected regions like eastern Congo [5,28-30] and include attitudes of blaming the victim, exonerating the rapist, and stigmatizing and rejecting the victim [29,31]. Stigmatization, in particular, has been shown to have a large impact on the wellbeing of sexual violence victims [32-34].

Sexual violence, and the social reactions it elicits, can possibly be framed within broader sociocultural perspectives on gender [18]. In DRC, as in many countries, gender norms and discourses are generally described as being supportive of the idea that women should be powerless and submissive [2,35,36], and should fulfill men’s sexual needs. This kind of sociocultural gender norm, which promotes unequal gender standards, may justify violence against women [37,38] and even lower the psychological threshold for sexual violence [2].

These norms also influence how victims often do not label their experiences of sexual violence as rape, because they perceive such coercion as normal [38] even if it complies with all legal definitions of rape. Equally, experiences of sexual violence often remain undisclosed because of fear of stigma, blame, or additional violence [39-41]. Additionally, a predominant ‘rape as a weapon of war’ discourse may instill particular stereotypes of sexual violence. As such, rape may be related to acts of sexual violence that are committed by strangers [42] or the military. Hence, victims might feel constrained from labeling an experience of sexual violence as rape or from seeking support if the sexual violence does not fit these stereotypes [43-46].

In developing countries, where young people rarely affirmatively answer general questions on ‘forced sex’ or ‘rape’, but still do experience a high prevalence of diverse forms of sexual violence (e.g. forced sex within marriage, attempted rape or fondling, exchange or transactional sex, forced prostitution), the concept of ‘non-consensual sexual experience’ , defined as a range of behaviors that includes unwanted penetrative sex, attempted rape, unwanted touch, and non-contact forms of abuse, is more often used [38,47]. Factors influencing whether young people label a non-consensual experience as rape are related to existing stereotypes of ‘typical rape’ , the nature of acquaintance or relationship with the perpetrator, the perpetrator’s behavior or level of intoxication, and the labeling of coercion as a normal sexual act [44,48,49].

Still, these forms of non-consensual sexual experience, which girls and young women endure but do not label as rape, may also have considerable mental and physical health consequences [50,51]. These may possibly differ from the impact of acknowledged rape experiences. Some studies have found that women who label experiences of sexual violence as rape tend to show less negative psychological consequences [52]. Moreover, they recover more quickly, since they are able to ‘redefine’ their experience [53], they reduce their feelings of self-blame [54], and more often seek support from others [55,56]. By contrast, Layman and colleagues (1996) [57] found that self-reported rape victims develop more posttraumatic stress symptoms.

However, most studies of sexual violence in war and armed conflict, in particular those conflict situations where sexual violence was/is used on a wide scale, do not differentiate between rape and non-consensual sexual violence, and only focus on self-reported rape. Additionally, little is yet known about the associations between daily stressors and stigmatization, victims’ own labeling of the sexual violence, and adolescents’ psychological wellbeing. This research therefore investigates the psychological sequelae of sexual violence in adolescent girls in war-affected eastern DRC in relation to their experiences of daily stressors and stigmatization and to their own labeling of the sexual violence they underwent (i.e. as ‘rape’ or as ‘non-consensual sexual experience’).

## Methods

### Participants and procedure

The study was conducted in the district of Ituri, eastern DRC, a region afflicted by armed conflict for decades [4,58,59]. Twenty-two secondary schools in all 10 neighborhoods across the large region of Ituri’s main city, Bunia, were randomly selected using stratified sampling in relation to location (rural, suburban, and urban regions); none of the selected schools refused to participate. In all schools, all female pupils in the second and third years of high school, where literacy and comprehension of the questionnaires could be assumed, were invited and consented to take part in the study (n = 1,304). Of the participants, who were aged 11 to 23 with a mean age of 15.89 (SD = 1.54) years old, 14.0% (n = 183) confirmed having been raped, while 24.2% (n = 315) mentioned having experienced a non-consensual sexual experience (NCSE) that they didn’t label as rape (Table 1). Some sociodemographic differences were found between the three groups of sexual violence (i.e. rape, non-consensual sexual experience, and no sexual violence) (Table 1).

**Table 1:** Socio-demographic characteristics of participants

	**Total group (n = 1,304)**	**No sexual violence (n = 806)**	**Rape (n = 183)**	**Non-consensual sex (n = 315)**	***F/χ***^***2***^
**Age**^†^	15.89 (1.54)	15.73 (1.49)	16.34 (1.51)	16.04 (1.63)	13.90*
**Socio-economic status**					21.076*
Bric house	600 (46.4%)	404 (50.5%)	86 (47.5%)	110 (35.3%)	
Non bric house	693 (53.6%)	396 (49.5%)	95 (52.5%)	202 (64.7%)	
**Parental availability**					22,10*
Both parents alive	991 (74.9%)	622 (78.2%)	112 (61.5%)	232 (75.1%)	
One or both parents deceased	333 (25.2%)	173 (21.8%)	70 (38.5%)	77 (24.9%)	

N(%); ^†^Mean (SD); *p < .01.

The questionnaires were administered in a six week period in 2011, during a 60- to 90-minute class period while the boys of the respective classes were engaged in other activities organized by the teacher. A description of the study was provided to the participants and followed by obtaining written informed consent from each. During the completion of the self-report questionnaires, the researcher or at least two research assistants were present to provide supervision and guidance. Questionnaires were administered in French, since this is the official language in secondary schools and a pilot study showed that students preferred French questionnaires over translated Kiswahili versions. Questionnaires were self-administered while thoroughly guided and structured by the research assistants. To promote inter-researcher reliability, extensive theoretical and practical training was provided to all research assistants (90 h). The researcher provided her contact details to participants, and also information on local psychological support projects for those in need of further professional care. The researcher had access to a large network of professional psychosocial services, which was used for referral of study participants. Ethical approval for the study was given by the Ethical Committee of the Faculty of Psychology and Educational Sciences, Ghent University.

### Measures

Six self-report questionnaires, all adapted for use in eastern Congo [60] were administered. First, a sociodemographic questionnaire investigated variables such as age, housing situation (as an indicator of participants’ socioeconomic status), and parental availability.

Second, the Adolescent Complex Emergency Exposure Scale (ACEES) [60] measured exposure to 14 context-specific and potentially traumatic war-related events (*yes/no)*, such as having witnessed people being killed, being separated from family, and having witnessed rape. Specific questions regarding experiences of sexual violence were added to the questionnaire. Besides the question ‘Have you experienced rape?’ , these comprised four questions referring to other forms of sexual violence or coercive sexual experience: being forced to have sex with a boyfriend, to have sex with someone you know, to have sex in exchange for goods, and to marry. These four forms of coercive sexual experience are, amongst others forms, mentioned as being “sexual violence” in 2006 Congolese legislation [61], and were considered by our Congolese expert committee guiding this study as the most relevant forms for the adolescents we wanted to study.

Third, the Adolescent Complex Emergency Daily Stressors Scale (ACEDSS) [60] inquired whether a range of different daily and social stressors (stigmatization) had occurred during the past month (*yes/no)*. They comprised 14 daily stressors (e.g., lack of food or medical care) and 14 stigmatization items (perceived discrimination and social exclusion in the familial and community context, e.g., being treated as if you were different, being isolated by the nuclear family, being treated badly by family members). These stigmatization items were initially derived from the Everyday Discrimination Scale [62] and then adapted to the particular cultural context following the procedure of Mels and colleagues [60].

Fourth, symptoms of posttraumatic stress were measured with the culturally adapted Congolese (French) version [60] of the Impact of Event Scale-Revised [63], a diagnostic self-administered questionnaire comprising 22 questions to be answered on a 5-point Likert scale (from 0 *never* to 5 *extremely*), accompanied by a visual probe. Items were grouped into three subscales (symptoms of intrusion, avoidance, and hyper-arousal). Cronbach’s alphas in this study were between .77 and .83.

Finally, the culturally adapted Congolese (French) version [60] of the Hopkins Symptom Checklist-37 for Adolescents [64] measured symptoms of anxiety (12 items), depression (13 items), and externalising problems (12 items). All items had to be answered on a 4-point Likert scale (from 1 *not/never* to 4 *always*), accompanied by a visual probe. Chronbach’s alphas in this study were between .60 and .85. The externalizing scale that had a Chronbach alpha of .60 was omitted from further analysis owing to insufficient reliability.

### Analysis

Descriptive statistics, ANOVA and *χ*^2^ analyses (with reports on odds ratios) were used to explore differences in sociodemographic characteristics, traumatic experiences, and daily and social stressors between the three types of sexual violence (victims of rape (Rape), victims of non-consensual sex (NCS) and those who did not report any of either (NSV)). ANOVA analyses were performed for total scores of traumatic exposure, daily stressors, and stigmatization as dependent variables, and ‘type of sexual violence’ as the independent variable, using dummy coding with base level ‘NSV’. The regression coefficients obtained indicate the expected mean differences between Rape and NCS compared to NSV.

Second, five separate ANCOVA analyses investigated associations between four factors – sociodemographic characteristics (age, socioeconomic status (brick house or other), parental availability (both parents alive or not)), traumatic exposure (total ACEES score), daily stressors and stigmatization (ACEDSS subscales), and type of sexual violence (NSV, NCS and Rape) – and mental health measures (HSCL-37A depression and anxiety subscales, IES-R intrusion, avoidance, and hyper-arousal subscales). All main terms were included in the model, as also the two-way interaction terms for type of sexual violence with the covariates ACEES and ACEDSS. To avoid multicollinearity, factors were effect-coded and the covariates were centered. Five separate models were fitted for the different mental health measures. Considering the large sample and the range of variables included in the analysis, the level of significance was put more conservatively at .01 to avoid Type I errors. Analyses were performed using R-2.15.2 [65].

## Results

### Stressful war events, daily stressors and stigmatization

Potentially traumatic war-related events were frequently reported (Table 2). Overall, victims of sexual violence (both rape and non-consensual sexual violence) were at higher risk of having experienced war-related traumatic events than girls who did not report sexual violence. Further the risk for victims of rape to have also experienced other traumatic events was higher than for participants who reported experiences of non-consensual sexual violence. Being enlisted as a child soldier and imprisonment were, in particular, frequently reported by rape victims.

**Table 2:** War-related traumatic experiences (ACEES)

	**Total (n = 1,304)**	**NSV (n = 806)**	**Rape (n = 183)**	**NCS (n = 315)**	**OR RAPE/NSV**	**OR NCS/NSV**	**OR Rape/NCS**	***χ***^***2***^ ***/F***^**†**^
Have been separated from family	284 (22.1%)	137 (17.2%)	50 (27.8%)	97 (31.2%)	1.85**	2.18**	0.85	29.38**
Have witnessed violent acts against family members or friends	172 (13.4%)	84 (10.5%)	37 (20.3%)	51 (16.9%)	2.17**	1.72**	1.26	16.29**
Had family members or friends violently killed during the war	515 (40.6%)	292 (37.3%)	96 (53.3%)	127 (41.5%)	1.92**	1.19	1.61	15.76**
Experienced pillage or setting your house on fire	592 (47.1%)	335 (43.1%)	116 (65.9%)	141 (46.4%)	2.56**	1.14	2.23**	30.15**
Experienced gunfire attacks	525 (42.3%)	276 (35.7%)	106 (59.6%)	143 (49.1%)	2.65**	1.74**	1.52	41.06**
Have seen somebody being killed	479 (38.1%)	245 (31.0%)	79 (51.3%)	155 (49.7%)	2.34**	2.20**	1.07	45.92**
Have seen dead or mutilated bodies	491 (38.4%)	250 (31.5%)	108 (59.7%)	133 (43.5%)	3.21**	1.67**	1.92*	53.79**
Have been injured during the war	113 (8.9%)	36 (4.5%)	41 (26.3%)	36 (11.5%)	7.56**	2.75**	2.74**	79.69**
Have been in prison	74 (5.7%)	6 (7.0%)	48 (26.7%)	20 (6.4%)	48.18**	9.11**	5.29**	183.22**
Have been enrolled in an armed group	48 (3.8%)	3 (.4%)	25 (16.0%)	20 (6.4%)	50.76**	18.22**	2.79*	95.73**
Have been kidnapped by an armed group	85 (6.5%)	16 (2.0%)	51 (28.0%)	18 (5.7%)	19.17**	2.99**	6.40**	165.01**
Have been forced to kill, injure or rape someone themselves	75 (5.9%)	25 (3.3%)	35 (22.2%)	14 (4.6%)	8.45**	1.42	5.96*	85.67**
Have seen someone being raped	180 (14.1%)	67 (8.4%)	56 (31.6%)	57 (18.9%)	5.08**	2.56**	1.98**	72.79**
*Total traumatic exposure (mean, SD)*	2.83 (2.43)	2.19 (1.90)	4.71 (3.05)	3.16 (2.45)	2.43**	1.01**	1.42**	86.62**

N(%); *p < .01, **p < .001; OR: odds ratio, NSV: no sexual violence, NCS: Non-consensual sexual experience.

Material daily stressors were equally frequently reported, with significant differences between the three groups (Table 3). Compared to participants who did not report sexual violence, victims of rape reported over eight times more risk of being accused of witchcraft and over four times more risk of insufficient medical care or lack of schooling. But girl victims of non-consensual sexual violence also showed a higher risk of experiencing daily stressors compared to girls who did not experience sexual violence. In addition, the results showed that girls who labeled their experiences as rape had a considerably higher risk of reporting daily stressors compared to adolescent victims of non-consensual sexual violence.

**Table 3:** Daily stressors (ACEDSS)

	**Total (n = 1,304)**	**NSV (n = 806)**	**Rape (n = 183)**	**NCS (n = 315)**	**OR Rape/NSV**	**OR NCS/NSV**	**OR Rape/NCS**	***χ***^***2***^ ***/F***^**†**^
Feelings of insecurity	522 (40.0%)	261 (33.3%)	100 (55.2%)	150 (48.1%)	2.47**	1.86**	1.33	40.72**
Impossibility to pay school fees	609 (46.4%)	313 (39.7%)	113 (61.7%)	171 (54.8%)	2.45**	1.84**	1.33	40.55**
Insufficient food	488 (37.7%)	230 (29.4%)	105 (59.0%)	142 (45.8%)	3.45**	2.03**	1.70*	65.98**
Insufficient clothing	529 (40.9%)	276 (35.5%)	95 (52.8%)	145 (46.8%)	2.03**	1.60**	1.27	24.25**
Sickness in family	778 (60.1%)	438 (56.1%)	127 (70.6%)	193 (62.7%)	1.88**	1.31	1.43	14.19*
Insufficient medical care	429 (33.1%)	201 (25.6%)	107 (60.5%)	112 (33.1%)	4.41**	1.66**	2.66**	81.73**
Skipping school because of working	364 (28.0%)	151 (19.2%)	94 (52.8%)	110 (35.7%)	4.72**	2.34**	2.01**	94.23**
Worried about family	773 (59.5%)	454 (57.7%)	95 (53.1%)	205 (66.6%)	0.83	1.46	0.57*	10.43*
High task load	508 (39.6%)	250 (32.0%)	116 (64.8%)	131 (43.8%)	3.91**	1.66**	2.36**	68.64**
Physical punishment	450 (34.9%)	226 (28.9%)	108 (60.7%)	112 (36.4%)	3.79**	1.40	2.70**	64.25**
Pursued by bad fate/bad spirits	329 (25.4%)	121 (15.4%)	112 (61.5%)	91 (29.6%)	8.79**	2.32**	3.80**	169.83**
Being sick	708 (54.7%)	399 (51.0%)	123 (68.0%)	176 (57.6%)	2.03**	1.29	1.57	18.52**
Living with too many people in a home	685 (52.8%)	366 (46.7%)	129 (71.3%)	178 (57.6%)	2.83**	1.55**	1.83*	39.14**
Don’t know father	252 (19.2%)	77 (9.6%)	107 (58.8%)	65 (20.9%)	13.41**	2.48**	5.40**	231.53**
*Total daily stressors (mean, SD)*	5.34 (3.31)	4.57 (2.98)	7.78 (2.98)	6.08 (3.11)	3.21**	1.51**	1.70**	201.12**

N(%); *p < .01, **p < .001; OR: odds ratio, NSV: no sexual violence, NCS: Non-consensual sexual experience.

Similarly, victims of sexual violence (both rape and non-consensual sexual violence) had a much higher risk of experiencing stigmatization compared to participants who did not report sexual violence (Table 4). Girls who reported rape experienced more stigmatization than girls who reported non-consensual sexual violence.

**Table 4:** Stigmatization (ACEDSS)

	**Total (n = 1,304)**	**NSV (n = 796)**	**Rape (n = 180)**	**NCS (n = 311)**	**OR Rape/NSV**	**OR NCS/NSV**	**OR Rape/NCS**	***χ***^***2***^ ***/F***^**†**^
Corporal punishment by family member	321 (24.3%)	79 (9.9%)	104 (56.8%)	132 (42.3%)	12.03**	6.70**	1.80*	251.00**
Hear that people say bad things about you or your family	577 (43.9%)	294 (36.8%)	113 (62.8%)	156 (50.0%)	2.90**	1.72**	1.69*	47.16**
You are treated worse than other people	326 (25.1%)	122 (15.4%)	109 (60.9%)	87 (28.2%)	8.55**	2.16**	3.96**	164.21**
You are treated with less respect than other people	370 (28.4%)	145 (18.2%)	104 (58.1%)	115 (37.3%)	6.22**	2.67**	2.33**	130.14**
You are treated badly by a family member	292 (22.3%)	102 (12.8%)	94 (51.6%)	88 (28.5%)	7.28**	2.71**	2.68**	139.89**
Rejected/abandoned by your (close) family	174 (13.3%)	44 (5.5%)	52 (28.7%)	73 (23.7%)	6.92**	5.35**	1.29	113.00**
Rejected/abandoned by your community	194 (14.8%)	44 (5.5%)	90 (49.5%)	57 (18.6%)	16.87**	3.93**	4.29**	232.29**
Treated as if people are scared of you	237 (18.1%)	63 (7.9%)	94 (52.2%)	75 (24.5%)	12.79**	3.80**	3.37**	206.81**
Threatened by others	428 (32.7%)	203 (25.5%)	99 (54.7%)	116 (37.7%)	3.53**	1.78**	1.98**	68.84**
Called dishonest	299 (22.8%)	107 (13.4%)	97 (53.6%)	91 (29.4%)	7.48**	3.73**	2.74**	149.25**
People act as if they’re better than you	612 (46.6%)	323 (40.5%)	120 (67.0%)	155 (49.7%)	2.99**	1.45	2.06**	43.27**
People act as if they’re smarter than you	584 (44.9%)	301 (38.1%)	112 (62.9%)	157 (51.0%)	2.75**	1.68**	1.63	43.37**
You receive poorer service than other people at stores/services	311 (23.8%)	147 (18.6%	71 (39.2%)	85 (27.6%)	2.83	1.67**	1.69*	38.34**
You are insulted	583 (44.5%)	299 (37.6%)	119 (65.7%)	155 (49.7%)	3.18	1.64**	1.94*	51.75**
*Total stigmatization (mean, SD)*	3.95 (3.45)	2.82 (2.50)	7.53 (4.28)	4.90 (3.37)	4.78	2.21**	2.63**	200.33**

N(%); *p < .01, **p < .001; OR: odds ratio, NSV: no sexual violence, NCS: Non-consensual sexual experience.

### Mental health

Mental health outcomes for the three groups are reported in Table 5. ANCOVA analyses investigating the impact of several independent variables on mental health outcomes revealed that participant’s socioeconomic status had no impact on mental health symptoms. Participants whose parents were still alive reported lower symptoms of anxiety (HSCL-37A).

**Table 5:** Mental health symptoms (IES-R, HSCL-37A)

	**No sexual violence (n = 806)**	**Rape (n = 183)**	**Non-consensual sex (n = 315)**
**IES-R**			
Avoidance/Numbing	1.80 (.70)	2.06 (.68)	2.14 (.76)
Intrusion	1.71 (.63)	1.83 (.69)	2.09 (.79)
Hyper-arousal	1.71 (.68)	2.08 (.67)	2.15 (.83)
Total score	1.74 (.61)	1.98 (.57)	2.12 (.71)
**HSCL-37A**			
Depression	1.61 (.33)	1.76 (.36)	1.77 (.37)
Anxiety	1.71 (.37)	1.79 (.37)	1.85 (.38)

Mean (SD).

War-related traumatic exposure (ACEES) impacted all mental health outcomes. Daily stressors were associated with an increase of posttraumatic stress symptoms (avoidance, intrusion, and hyper-arousal). The level of stigmatization was associated with symptoms of depression and anxiety (HSCL-37A) and hyper-arousal (IES-R). Hereby, higher scores in war-related trauma, stigmatization, and daily stressors always led to more psychological problems.

**Figure 1:**
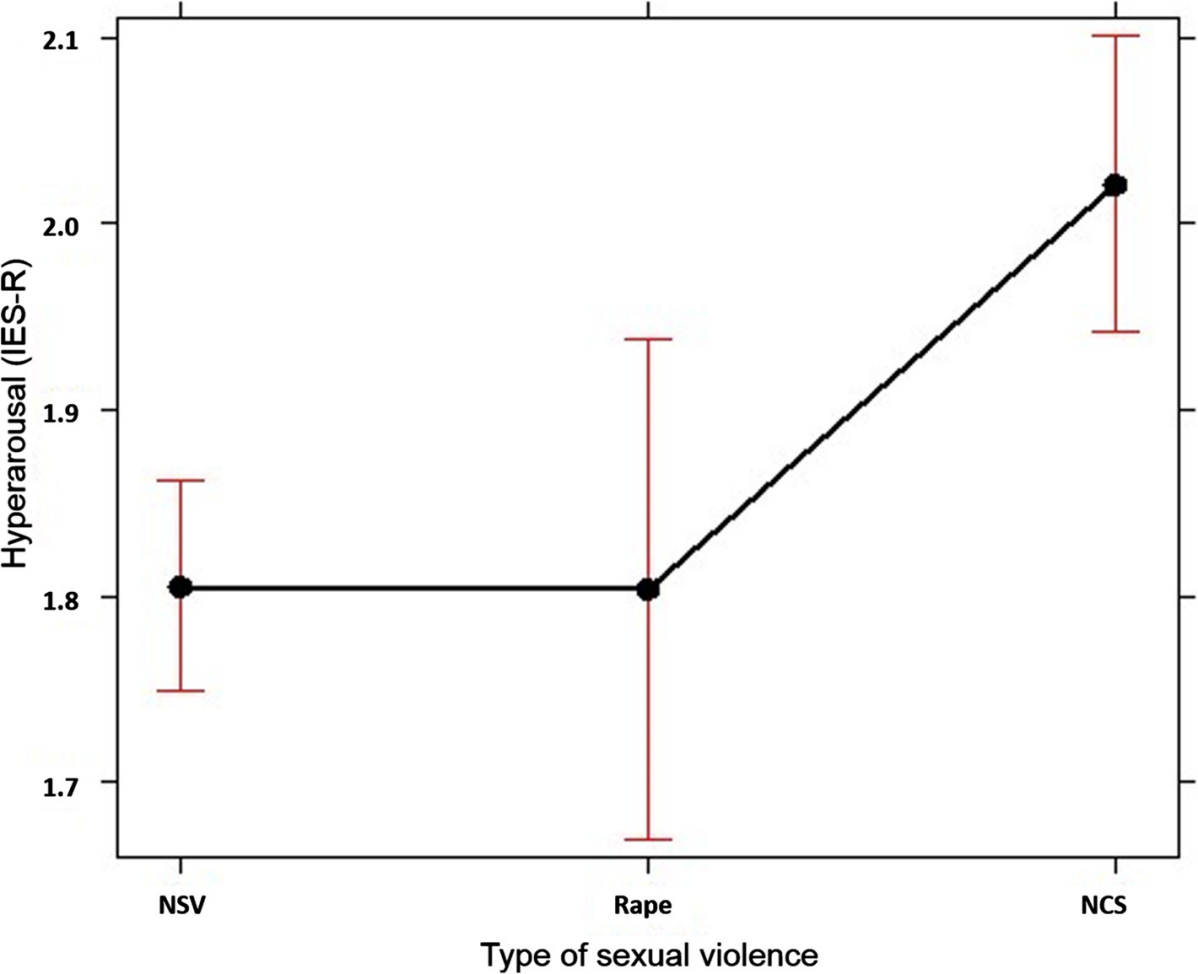
**Estimated means and point-wise 95% confidence bands of type on IES-R hyper-arousal symptoms.**

**Figure 2:**
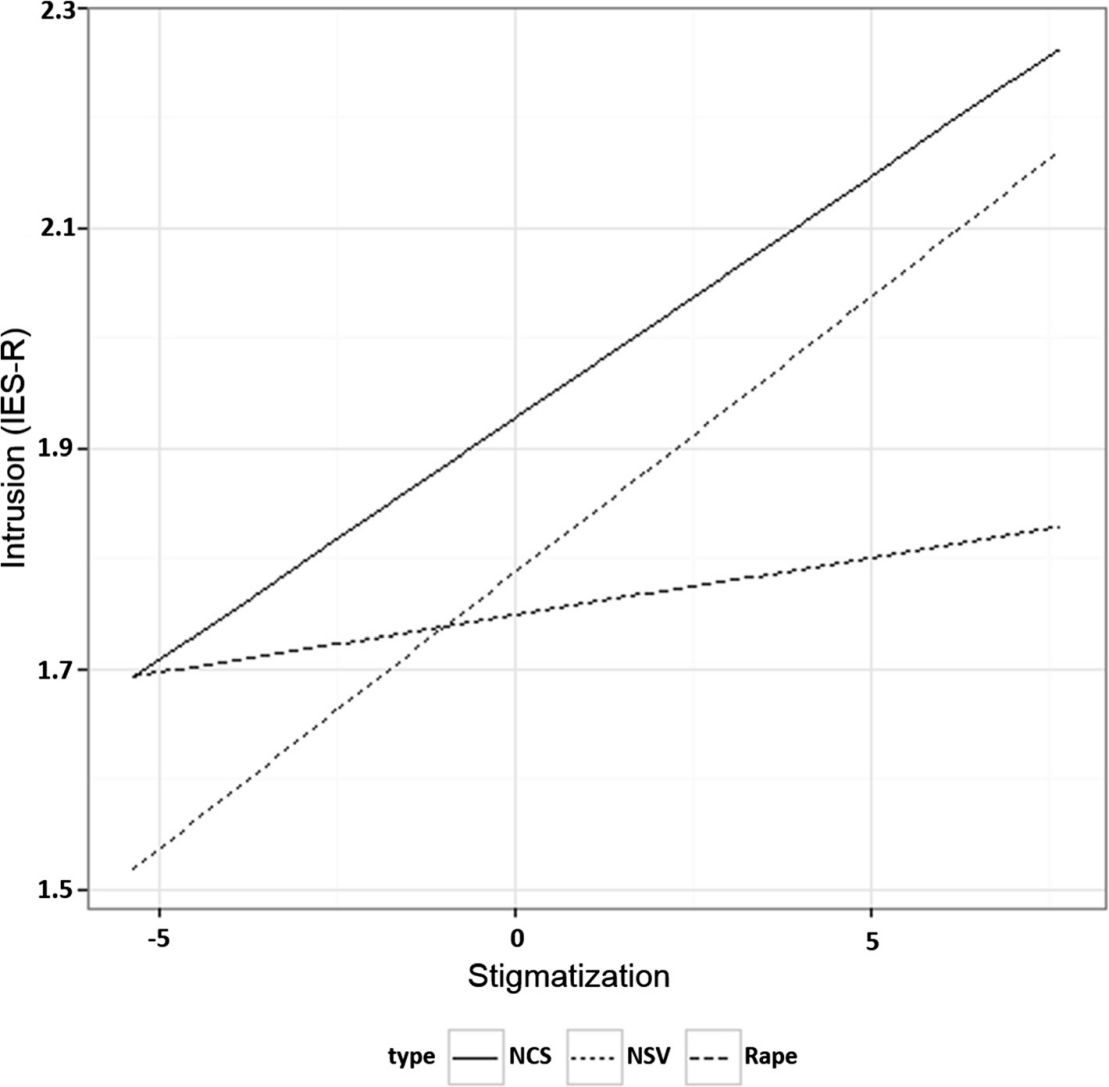
Interaction effect type of sexual violence and stigmatization on IES-R intrusion symptoms.

The type of sexual violence that participants reported impacted hyper-arousal symptoms (see also additional variance explained by type of sexual violence, as mentioned in Table 6). The highest level was for girls who reported experiences of non-consensual sexual violence, although we need to take into account the high variance of hyper-arousal symptoms reported by girls who labeled their sexual violence experiences as rape (Figure 1). For symptoms of intrusion (IES-R), an interaction effect between type of sexual violence and the covariate stigmatization was found (Figure 2). All types of sexual violence showed positive relations between stigmatization and intrusion symptoms, but compared to girls with rape experiences, the estimated regression lines showed stronger effects for participants who experienced non-consensual sexual violence or who did not report sexual violence, with higher mean scores for victims of non-consensual sexual violence. The estimated regression line for victims of rape thus indicated that the expected level of intrusion symptoms depended less on the level of stigmatization than for the other groups. A similar interaction effect was also found for daily stressors and type of sexual violence on symptoms of depression and anxiety: expected levels of anxiety and depression were less dependent on daily stressors for rape victims, while a strong positive relation was found for girls report NCSE or no sexual violence.

**Table 6:** ANCOVA analysis investigating the impact of different variables on mental health outcomes

	**Avoidance**	**Intrusion**	**Hyper-arousal**	**Depression**	**Anxiety**
(Intercept)	1.76**	1.79**	1.86**	1.61**	1.89**
Age	.01	.00	.00	.01	-.01
Socio-economic status	-.03	-.03	-.04	-.02	-.01
Parental availability	-.03	-.02	-.05	-.05	-.04*
Type of sexual violence	(−.04)(−.03)	(−.02)(−.10)	(−.04)(−.10)	(.01)(−.04)	(.02)(−.06)
	F(2,1228) = 3.06	F(2,1260) = 6.00*	F(2,1257) = 9.15**	F(2,1248) = 2.35	F(2,1251) = 2.76
Traumatic exposure	.07**	.09**	.09**	.03**	.03**
Daily stressors	.04**	.04**	.05**	.01**	.02**
Stigmatization	.01	.02*	.03**	.02**	.01**
Type of sexual violence * traumatic exposure	(.01)(−.02)	(.00)(−.03)	(.01)(−.01)	(.00)(.00)	(.00)(.00)
	F(2,1228) = 0.86	F(2,1260) = 3.37	F(2, 1257) = 0.63	F(2, 1248) = 0.07	F(2, 1251) = 0.16
Type of sexual violence * daily stressors	(.01)(−.02)	(.01)(−.02)	(.01)(−.02)	(.01)(−.02)	(.00)(−.02)
	F(2,1228) = 1.94	F(2,1228) = 1.88	F(2, 1257) = 1.21	F(2, 1248) = 4.98*	F(2, 1251) = 4.82*
Type of sexual violence * stigmatization	(.02)(−.02)	(.00)(−.04)	(−.01)(−.01)	(.01)(−.00)	(.01)(−.01)
	F(2,1228) = 1.94	F(2,1260) = 8.87**	F(2, 1257) = 1.46	F(2, 1248) = 1.28	F(2, 1251) = 4.22
Explained variance without impact of type of sexual violence (r^2^)	17.7%	20.7%	27.3%	21.3%	14.2%
Overall explained variance (r^2^)	19.9%	27.9%	30.2%	24.0%	18.0%

For dichotomous variables: β; Estimated Regression Coefficients, SE: Standard error; For non-dichotomous variables: (β1)(β2) for effect-coded variables for factor sexual violence and F(df1, df2); *p < .01, **p < .001.

## Discussion

More than one third of the eastern Congolese girls in this study reported experiences of sexual violence, which is congruent with the high prevalence of sexual violence in eastern Congo that has been reported for many years [11]. Girls who lived in poorer socioeconomic situations, as well as girls who have lower parental availability (one or two parents deceased), reported more sexual violence experiences than their peers. While little research has been undertaken in this context, other studies alluded to the risk that both a poorer socioeconomic status and parental separation could lead to a higher prevalence of sexual violence [2]. On the other hand, sexual violence could in its turn also lead to more difficult economic situations or to separation from parents (exclusion from family).

It is noteworthy, however, that about 63% of the girls who reported sexual violence did not label this experience as rape, although legally, their experiences of sexual violence qualified as such [66]. This was probably engendered by the strong current discourses describing rape in Congo as a ‘weapon of war’ , thereby seemingly silencing rape by civilians or other forms of sexual violence (e.g. forced sexual experiences in marriage), which are described as ‘everyday, and even boring’ [6]. Particular sociocultural gender norms about rape may also have restrained victims from labeling their sexual violence experiences as rape because of either uncertainty as to whether or not an experience was rape [67] or fear of stigmatization [39].

This latter element shows up as highly relevant in our study, since girls who label their experiences as rape are clearly confronted with more negative social responses, as shown in the high levels of reported stigmatization experiences. We hereby need to acknowledge that these experiences of stigmatization referred to *perceived* stigmatization, which might be higher in particular conceptualizations of sexual violence (i.e. when the sexual violence is labelled as ‘rape’), linked to certain expectancy effects with regards to stigmatisation.

Next to these higher levels of stigmatization, for both war-related traumatic experiences and material daily stressors we found similar patterns as for stigmatization: rape victims clearly reported more of these compared to victims who reported non-consensual sexual violations, although the latter group also experienced more of all investigated stressors (war-related trauma, daily stressors, and stigmatization) compared to girls who did not report any sexual violence experience. This clearly shows that sexual violence is mostly not a single trauma event, but goes hand in hand with exposure to other impacting events, whether concurrently with, preceding, or in the aftermath of the sexual violence, the latter being particularly the case with stigmatization of the victim by the wider community [5,28-30].

This accumulation of stressors and traumatic events is one of the greatest explanatory factors in the mental health impact of sexual violence on adolescent victims, as shown in the high prevalence of mental health problems in this study, both in participants who labeled the sexual violence as rape as those who did not [52,57,68]. However, contrary to the expectation that – given the lower exposure to all investigated stressors in victims of non-consensual sexual violence compared to rape victims and the high explanatory role of these factors in mental health outcomes – the latter group would report significantly higher levels of mental health symptoms, we did not find significant differences between the two groups in symptoms of posttraumatic stress (avoidance), depression and anxiety. And for hyperarousal and intrusion symptoms (PTSD), we even found higher levels in girls who did not label their sexual violence experience as rape, in this respect also differing from other studies that reported higher levels of PTSD symptoms in acknowledged compared to unacknowledged rape victims [52]. Possible explanations for this interesting finding could be that, as suggested in the literature, the acknowledgement of non-consensual sexual experiences as rape allows the victim to ‘redefine’ the experience and/or to reduce feelings of self-blame [53,54], two mechanisms that can lower the mental health impact of the traumatic events [40]. The evidence suggests, although not consistently, that victims of sexual violence who do not label their sexual violence experience as rape report more impaired coping [52], which is in its turn associated with negative mental health outcomes after a traumatic experience [41,69]. Another hypothesis is that the elevated negative social reactions of the community to adolescents’ experiences of rape could induce these girls to cope more actively so as to ensure their wellbeing, including possibly also to search more for (other) sources of social support [56]. Although further study is required to investigate these findings, it is clear that the labeling of the sexual violence as ‘rape’ or as ‘non-consensual sexual experience’ has important associations with the exposure of the adolescent girl victim to other stressors (war-related trauma, daily stressors and stigma) and with the mental health consequences of these impacting experiences.

### Implications

This study has important clinical implications. First, sexual violence has a large negative impact on adolescents’ mental health [19-21]. While the need for psychosocial support to acknowledged rape victims has been recognized (although often it is not readily available), this study clearly describes the need for mental health support to victims of sexual violence who do not label their experience as rape. Sensitization campaigns to raise awareness of a broadened definition of sexual violence, including other forms of non-consensual sexual experience, and the large negative impact of these experiences, could be a first step towards also reaching out to these adolescents and offering them – where needed and wished – appropriate support services. It is also necessary to address victims’ possible feelings of shame and self-blame, working towards ways to redefine – where needed – the events that took place.

Second, the large prevalence of stigmatization, and its severe impact on victims’ mental health point towards context-oriented interventions. These need to be threefold. First, in psychosocial support offers to adolescent victims, significant context figures (family members, friends, community members) need to be involved [27,70] so as to reduce stigmatization mechanisms in the adolescent’s immediate context and to increase social support sources [24]. Hereby, attention should be paid towards possible differences between perceived and actual or intended experiences of stigmatization. Second, on a community and society level awareness and sensitization campaigns are needed to address the problem of sexual violence and its consequences. Community perceptions and attitudes towards rape, including stigmatization and blaming attitudes, influence how victims of sexual violence label their experiences, impact their mental health [32-34], and restrain adolescents, in particular those who label their experiences as non-consensual sexual experiences, from disclosing them and seeking help [39,71]. Hereby, it could be helpful to supersede particular narrow definitions of sexual violence (e.g., with labels such as ‘rape’), and to promote a broader construct, such as ‘sexual or gender-based violence’. Lastly, these sensitization and awareness campaigns also hopefully help to reduce the prevalence of sexual violence, since its prevalence in particular post-conflict contexts is still astonishing. These sensitization activities could address stereotypes of sexual violence and its consequences with the aim of promoting acknowledgment of rape by individuals and of fostering positive social reactions in order to create a supportive social environment [24,71,72]. While often interventions on sexual violence and non-consensual sexual experiences target girls in particular, public health programming seems to be needed for both men and women, on traditional gender roles and norms, and on perceptions of non-consensual sexual experiences [38].

Overall, an integrative and ecological approach towards psychosocial support for adolescent victims of sexual violence is needed, including not only interventions with the individual victim, but also interventions directed towards the wider social ecology of the adolescents.

### Limitations

The interpretation of the study’s findings needs to consider the following limitations. First, although we included several questions in an effort to include different types of sexual violence (rape, several forms of non-consensual sexual experiences), the figures about the prevalence of sexual violence in this study might still be an underestimation of reality, due to fear of accusation or stigma, due to the ongoing insecurity in the region, due to particular emotional connotations possibly linked to the word ‘rape’ , or due to the ways the questions on sexual violence (and the examples given) were framed [2,71]. This implies that some adolescents in the ‘non-sexual-violence’ group could still have been victims of sexual violence. On the other hand, although this was clearly and repeatedly stressed during the research, particular expectations by participants of receiving material compensation for their participation or for particular answers might have influenced participants’ responses to increase their reporting of sexual violence experiences.

Second, this study also focuses solely on girls, while also considerable levels of sexual violence towards men and boys in the region have been reported [28]. This choice was made in close consultation with the local expert team guiding this study, given that boys’ responses might be highly influenced by taboos regarding the sexual violation of boys, rendering this method (self-report measures in a class-room setting) less applicable for boys. Also, for logistical reasons only girls who were in schools were included in the study, reducing its generalizability to out-of-school adolescents.

Third, while the psychological impact of acknowledgement of rape has been studied, there was no previous knowledge of the psychological well-being of the participants. These longitudinal findings could have added to an understanding of whether already existing psychological problems influenced the labeling of the sexual violence experiences as non-consensual sexual experience or as rape. Above, the impact of parental availability was only conceptualized as whether parents were still alive or not, which is possibly not related to parents’ emotional availability.

Last, the questionnaires could not cover all mental health problems, nor all participants’ experiences of trauma or stigmatization, although all questionnaires were rigorously adapted, both culturally and contextually, for use in this particular context.

## Conclusions

More than one third of eastern Congolese adolescent girls reported experiences of sexual violence. This study shows the large association of sexual violence with other stressors (daily stressors, stigmatization, and stressful war events), and the impact of all of these on the girl victims’ mental health. Girls who did not label their sexual violence experience as rape reported more posttraumatic intrusion and hyper-arousal symptoms compared to those labeling the sexual violence as rape. These findings point to the important association between labeling a sexual violence experience and mental health, in relation to other stressors, in particular daily stressors, war-related trauma, and stigmatization. Important implications of these findings are the need to implement an integrative and context-oriented approach towards psychosocial support for adolescent victims of sexual violence, thereby hopefully fostering an enhanced supportive social environment. Furthermore, culturally appropriate sensitization activities need to be developed for communities and other stakeholders in order to address stereotypes of sexual violence and its consequences and in an effort to reduce the prevalence of sexual violence and adverse social reactions to it.

## Abbreviations

ACEDSS: Adolescent complex emergency daily stressors scale; ACEES: Adolescent complex emergency exposure scale; ANCOVA: Analysis of covariance; DRC: Democratic Republic of Congo; IES-R: Impact of event scale-revised; HSCL-37A: Hopkins symptom checklist-37 for adolescents; NCS: Non-consensual sexual experience; NSV: No sexual violence; PTSD: Posttraumatic stress disorder.

## Competing interests

The authors declare that they have no competing interests.

## Authors’ contributions

AV and ID participated in the acquisition of data and revision of the manuscript. AV, ID, MDS conceived of the study, determined the design and provided in Administrative, technical or material support. AV, ID, MDS and EB performed the statistical analysis, interpreted the data, drafted the manuscript and provided a critical revision for important intellectual content. ID and EB obtained funding and provided supervision of the study. All authors read and gave final approval for the version submitted for publication.

## Pre-publication history

The pre-publication history for this paper can be accessed here:

http://www.biomedcentral.com/1472-6874/14/106/prepub

## References

[B1] Coghlan B Brennan R Ngoy P Dofara D Otto B Clements M Stewart T Mortality in the Democratic Republic of the Congo: a nationwide survey Lancet 2006 367 9504 44 51 10.1016/S0140-6736(06)67923-3 16399152

[B2] Dolan C War is not over yet: community perceptions of sexual violence and its underpinnings in Eastern DRC Int Alert 2010 http://www.international-alert.org/sites/default/files/publications/1011WarIsNotYetOverEng.pdf. [Accessed August 8, 2011]

[B3] Pottier J Displacement and ethnic reintegration in Ituri, DRCongo: challenges ahead J Mod Afr Stud 2008 46 3 427 450

[B4] Vlassenroot K Raeymaekers T The politics of rebellion and intervention in Ituri: the emergence of a new political complex? Afr Aff 2004 103 412 385 412 10.1093/afraf/adh066

[B5] Bartels S VanRooyen M Leaning J Scott J Kelly J “Now, the world is without me”: an investigation of sexual violence in Eastern Democratic Republic of Congo Harv Humanitarian Initiat Oxfam Int 2010 http://hhi.harvard.edu/sites/default/files/publications/hhi-oxfam%20drc%20gbv%20report.pdf. [Accessed July 20, 2010]

[B6] Eriksson-Baaz M Stern M Sexual Violence As A Weapon Of War? Perceptions, Prescriptions, Problems In The Congo And Beyond 2013 London: AfricaNow

[B7] Human Rights Watch Kosovo: Rape as a Weapon of “Ethnic Cleansing” 2000 New York; NY: Human Rights Watch

[B8] Leatherman J Sexual Violence And Armed Conflict 2011 Cambridge: Polity Press

[B9] Wood EJ Variation in sexual violence during war Polit Soc 2006 34 3 307 41 10.1177/0032329206290426

[B10] UN Security Council Security Council Resolution 1820 (2008) [On Acts Of Sexual Violence Against Civilians In Armed Conflicts] S/RES/1820 2008 http://www.unhcr.org/refworld/docid/485bbca72.html. [Accessed December 2, 2012

[B11] Peterman A Palermo T Bredenkamp C Estimates and determinants of sexual violence in the democratic Republic of the Congo Am J Public Health 2011 101 6 1060 1067 10.2105/AJPH.2010.300070 21566049 PMC3093289

[B12] Thomas DQ Ralph RE Ramet SP Rape In War: The Case Of Bosnia Gender Politics in the Western Balkans. Women and Society in Yugoslavia and the Yugoslav Successor States 1999 University Park, PA: Pennsylvania University Press 203 218

[B13] Human Rights Watch Soldiers Who Rape, Commanders Who Condone: Sexual Violence And Military Reform in the Democratic Republic of Congo 2009 New York: Human Rights Watch

[B14] Mukwege DM Nangini C Rape with extreme violence: the wew pathology in South Kivu, Democratic Republic of Congo PLoS Med 2009 6 e1000204 10.1371/journal.pmed.1000204 20027215 PMC2791171

[B15] Maedl A Rape as weapon of War in the Eastern DRC? The Victims’ perspective Human Rights Q 2011 33 128 147 10.1353/hrq.2011.0005

[B16] Kalisya LM Justin PL Kimona C Nyavandu K Eugenie KM Jonathan KML Claude KM Hawkes M Sexual violence toward children and youth in war-torn Eastern Democratic Republic of Congo PLoS One 2011 6 1 e15911 10.1371/journal.pone.0015911 21267467 PMC3022750

[B17] Nelson BD Collins L VanRooyen MJ Joyce N Mukwege D Bartels S Impact of sexual violence on children in the Eastern Democratic Republic of Congo Med Confl Surviv 2011 27 4 211 225 10.1080/13623699.2011.645148 22416569

[B18] Douma N Hilhorst D Fond De Commerce? Sexual Violence Assistance In The Democratic Republic Of Congo Occasional Paper no. 2 2012 Wageningen, The Netherlands: Wageningen University, Disaster Studies

[B19] Bartels S Scott J Leaning J Mukwege D Lipton R Van Rooyen M Surviving sexual violence in Eastern Democratic Republic of Congo J Int Women’s Stud 2010 11 4 4 37 49

[B20] Johnson K Scott J Rughita B Kisielewski M Asher J Ong R Lawry L Association of sexual violence and human rights violations with physical and mental health in territories of the Eastern Democratic Republic of the Congo JAMA 2010 304 5 553 562 doi:10.1001/jama.2010.1086 10.1001/jama.2010.1086 20682935

[B21] Resick PA The psychological impact of rape J Interpers Violence 1993 8 223 255 10.1177/088626093008002005

[B22] Bal S Crombez G Van Oost P Deboudeaudhuij I The role of social support in well-being and coping with self-reported stressful events in adolescents Child Abuse Negl 2003 27 12 1377 1395 10.1016/j.chiabu.2003.06.002 14644056

[B23] Allwood MA Bell-Dolan D Husain SA Children’s trauma and adjustment reactions to violent and nonviolent war experiences J Am Acad Child Adolesc Psychiatry 2002 41 4 450 457 10.1097/00004583-200204000-00018 11931602

[B24] Campbell R Dworkin E Cabral G An ecological model of the impact of sexual assault on women’s mental health Trauma Violence Abuse 2009 10 225 246 10.1177/1524838009334456 19433406

[B25] Ellis H MacDonald H Lincoln A Cabral H Mental health of Somali adolescent refugees: the role of trauma, stress and perceived discrimination J Consult Clin Psychol 2008 76 2 184 193 18377116 10.1037/0022-006X.76.2.184

[B26] Miller KE Omidian P Rasmussen A Yaqubi A Daudzai H Daily stressors, war experiences, and mental health in Afghanistan Transcult Psychiatry 2008 45 4 611 638 10.1177/1363461508100785 19091728

[B27] Miller KE Rasmussen A War exposure, daily stressors, and mental health in conflict and post-conflict settings: bridging the divide between trauma-focused and psychosocial frameworks Soc Sci Med 2010 70 7 16 10.1016/j.socscimed.2009.09.029 19854552

[B28] Johnson K Asher J Rosborough S Raja A Panjabi R Beadling C Lawry L Association of combatant status and sexual violence with health and mental health outcomes in postconflict Liberia JAMA 2008 300 6 676 690 10.1001/jama.300.6.676 18698066

[B29] Kelly J Kabanga J Cragin W Alcayna-Stevens L Haider S Vanrooyen MJ ‘If your husband doesn’t humiliate you, other people won’t’: gendered attitudes towards sexual violence in eastern Democratic Republic of Congo Glob Public Health 2012 7 3 285 298 10.1080/17441692.2011.585344 21660787

[B30] Kelly JT Betancourt TS Mukwege D Lipton R VanRooyen MJ Experiences of female survivors of sexual violence in eastern Democratic Republic of the Congo: a mixed-methods study Confl Heal 2011 5 25 http://www.conflictandhealth.com/content/5/1/25 [Accessed February 12, 2012] 10.1186/1752-1505-5-25 PMC3271036 22047181

[B31] Bohner G Pina A Viki Tendayi G Siebler F Using social norms to reduce men’s rape proclivity: perceived rape myth acceptance of out-groups may be more influential than that of in-groups Psychol Crime Law 2010 16 8 671 693 10.1080/1068316X.2010.492349

[B32] Betancourt TS Agnew-Blais J Gilman SE Ellis BH Past horrors, present struggles: the role of stigma in the association between war experiences and psychosocial adjustment among former child soldiers in Sierra Leone Soc Sci Med 2010 70 1 17 26 10.1016/j.socscimed.2009.09.038 19875215 PMC3756934

[B33] Campbell R Ahrens C Sefl T Wasco SM Barnes HE Social reactions to rape victims: healing and hurtful effects on psychological and physical health outcomes Violence Vict 2001 16 287 302 11437118

[B34] Ullman SE Starzynski LL Long S Mason G Long LM Sexual assault disclosure, social reactions, and problem drinking in women J Interpers Violence 2008 23 1235 1257 10.1177/0886260508314298 18309039 PMC3863580

[B35] Pankhurst D Pankhurst D Introduction: Gendered War And Peace Gendered War And Peace 2008 New York: Routledge 1 30

[B36] Puechgirbal N Women and war in the Democratic Republic of Congo Signs J Women Cult Soc 2003 28 4 1271 1281 10.1086/368319

[B37] Heise L Ellsberg M Gottemoeller M Ending violence against women Popul Rep 1999 11 http://www.k4health.org/sites/default/files/L%2011.pdf [Accessed Mai 6, 2011]

[B38] Jejeebhoy SJ Bott S Jejeebhoy SY, Shah I, Thapa S Non-Consensual Sexual Experiences Of Young People In Developing Countries Sex Without Consent: Young people in Developing Countries 2005 New York: Macmillan 3 46

[B39] Duroch F McRae M Grais RF Description and consequences of sexual violence in Ituri province, Democratic Republic of Congo BMC Int Health Human Rights 2011 11 5 http://www.biomedcentral.com/1472-698X/11/5 [Accessed July 16, 2012] 10.1186/1472-698X-11-5 PMC3108309 21504596

[B40] Arata CM Coping with rape: the roles of prior sexual abuse and attributions of blame J Interpers Violence 1999 14 62 78 10.1177/088626099014001004

[B41] Braun-Lewensohn O Celestin-Westreich S Celesing LP Verleye G Verté D Ponjaert-Kristoffersen I Coping styles as moderating the relationships between terrorist attacks and well-being outcomes J Adolesc 2009 32 585 599 10.1016/j.adolescence.2008.06.003 18775563

[B42] Kahn AS Mathie VA Torgler C Rape scripts and rape acknowledgment Psychol Women Q 1994 18 53 66 doi:10.1111/j.1471-6402.1994.tb00296.x 10.1111/j.1471-6402.1994.tb00296.x

[B43] Bondurant B University women’s acknowledgment of rape: individual, situational, and social factors Violence Against Women 2001 7 294 314 10.1177/1077801201007003004

[B44] McGee H O’Higgins M Garavan R Conroy R Rape and child sexual abuse: what beliefs persist about motives, perpetrators, and survivors? J Interpers Violence 2011 26 17 3580 3593 10.1177/0886260511403762 21859758

[B45] Peterson ZD Muehlenhard CL Was it rape? The function of women’s rape myth acceptance and definitions of sex in labeling their own experiences Sex Roles 2004 51 129 144

[B46] Ryan KM The relationship between rape myths and sexual scripts: the social construction of rape Sex Roles 2011 65 774 782 DOI 10.1007/s11199-011-0033-2 10.1007/s11199-011-0033-2

[B47] Erulkar AS The experience of sexual coercion among young people in Kenya. International family planning perspectives Gend Based Violence Reprod Health 2004 30 4 182 189 10.1363/3018204 15590384

[B48] Jewkes R Jejeebhoy SY, Shah I, Thapa S Non-Consensual Sex Among South African Youth: Presence of Coerced Sex and Discourse of Control and Desire Sex Without Consent: Young people in Developing Countries 2005 New York: Macmillan 86 95

[B49] Kahn AS Jackson J Kully C Badger K Halvorsen J Calling it rape: differences in experiences of women who do or do not label their sexual assault as rape Psychol Women Q 2003 27 233 242 10.1111/1471-6402.00103

[B50] Jejeebhoy SJ Shah I Thapa S Sex Without Consent: Young People in Developing Countries 2005 New York: Macmillan

[B51] Krug E Dahlberg L Mercy JA Zwi AB Lozano R World Report on Violence and Health 2002 Geneva Switzerland: WHO

[B52] Clements CM Ogle RL Does acknowledgment as an assault victim impact postassault psychological symptoms and coping? J Interpers Violence 2009 24 1595 1614 doi:10.1177/0886260509331486 10.1177/0886260509331486 19252070

[B53] Gidycz CA Koss MP Predictors of long-term sexual assault trauma among a national sample of victimized college women Violence Vict 1991 6 175 190 1818616

[B54] Botta RA Pingree S Interpersonal communication and rape: women acknowledge their assaults J Health Commun 1997 2 197 212 10.1080/108107397127752 10977247

[B55] Littleton HL Axsom D Breitkopf CR Berenson A Rape acknowledgment and postassault experiences: how acknowledgment status relates to disclosure, coping, worldview, and teactions received from others Violence Vict 2006 21 6 761 778 10.1891/0886-6708.21.6.761 17220018

[B56] Harned MS Understanding women’s labeling of unwanted sexual experiences with dating partners Violence Against Women 2005 11 374 413 10.1177/1077801204272240 16043555

[B57] Layman MJ Gidycz CA Lynn SJ Unacknowledged versus acknowledged rape victims: situational factors and posttraumatic stress J Abnorm Psychol 1996 105 1 124 131 8666701 10.1037//0021-843x.105.1.124

[B58] Punier G From Genocide to Continental War: The Congolese Conflict and the Crisis of Contemporary Africa: The Congo Conflict and the Crisis of Contemporary Africa 2009 London: C Hurst & Co Publishers Ltd

[B59] Human Rights Watch Ituri: “Covered in Blood”: Ethnically Targeted Violence in Northeastern DR Congo’ 2003 Washington, DC: HRW

[B60] Mels C Derluyn I Broekaert E A community-based procedure for the cross-cultural adaptation of mental health self-report measures in emergency settings: validation of the IES-R and HSCL-37A for use in Eastern Democratic Republic of Congo Soc Psychiatry Psychiatr Epidemiol 2010 45 899 910 10.1007/s00127-009-0128-z 19707700

[B61] DRC government La Loi Sur Les Violences Sexuelles 2006 Kinshasa: DRC government

[B62] Williams DR Yan Yu JS Anderson NB Racial differences in physical and mental health: socioeconomic status, stress and discrimination J Health Psychol 1997 2 335 351 10.1177/135910539700200305 22013026

[B63] Weiss D Keane T The Impact of Event Scale-Revised Assessing Psychological Trauma and PTSD 2004 New York: Guilford 168 189

[B64] Bean T Derluyn I Eurelings-Bontekoe E Broekaert E Spinhoven P Validation of the multiple language versions of the Hopkins symptom checklist-37 for refugee adolescents Adolescence 2007 42 165 51 71 17536475

[B65] R Development Core Team R: A Language And Environment For Statistical Computing 2011 Vienna, Austria: R Foundation for Statistical Computing URL http://www.R-project.org/

[B66] McMullin D White JW Long-term effects of labeling a rape experience Psychol Women Q 2006 30 96 105 10.1111/j.1471-6402.2006.00266.x

[B67] Koss MP Pirog-Good MA, Stets JE Hidden Rape: Sexual Aggression And Victimization In A National Sample Of Students In Higher Education Violence In Dating Relationships: Emerging Social Issues 1989 New York: Praeger 145 184

[B68] Koss MP Harvey MR The Rape Victim: Clinical and Community Interventions 1991 Thousand Oaks, CA: Sage

[B69] Paardekooper B de Jong J Hermanns J The psychological impact of war and the refugee situation on South Sudanese children in refugee camps in Northern Uganda: an exploratory study J Child Psychol Psychiatry 1999 40 529 536 10.1111/1469-7610.00471 10357160

[B70] Kelly J VanRooyen M Kabanga J Maclin B Mullin C Hope for the future again. Tracing the effects of sexual violence and conflict on families and communities in Eastern Democratic Republic of the Congo Harv Humanit Initiat 2010 http://hhi.harvard.edu/sites/default/files/publications/publications%20-%20women%20-%20hope.pdf. [Accessed July 20, 2010]

[B71] Flisher AJ Jejeebhoy SY, Shah I, Thapa S Non-Consensual Adolescent Sexual Experiences: Policy Implications Sex Without Consent: Young people in Developing Countries 2005 New York: Macmillan 269 285

[B72] Jejeebhoy SY Thapa S Jejeebhoy SY, Shah I, Thapa S Non-Consensual Sex And Young People: Looking Ahead Sex Without Consent: Young people in Developing Countries 2005 New York: Macmillan 3 46

